# Subclinical Cardiac Dysfunction in Children with Coeliac Disease: Is the Gluten-Free Diet Effective?

**DOI:** 10.5402/2012/706937

**Published:** 2012-11-14

**Authors:** Berna Saylan, Ayhan Cevik, Ceyda Tuna Kirsaclioglu, Filiz Ekici, Ozgur Tosun, Gonca Ustundag

**Affiliations:** ^1^Department of Pediatric Cardiology, Faculty of Medicine, Zonguldak Karaelmas University, 67600 Zonguldak, Turkey; ^2^Department of Pediatric Cardiology, Faculty of Medicine, Gazi University, 06500 Ankara, Turkey; ^3^Department of Pediatric Gastroenterology, Ankara Diskapi Children's Hospital, 06110 Ankara, Turkey; ^4^Department of Pediatric Cardiology, Ankara Diskapi Children's Hospital, 06110 Ankara, Turkey; ^5^Department of Biostatistics and Medıcal Informatics, Faculty of Medicine, Akdeniz University, 07059 Antalya, Turkey; ^6^Department of Pediatric Gastroenterology, Faculty of Medicine, Zonguldak Karaelmas University, 67600 Zonguldak, Turkey

## Abstract

*Objectives*. The aim of this study is to investigate the effects of coeliac disease on cardiac function in children using conventional transthoracic echocardiography (TTE) and tissue Doppler echocardiography (TDE). *Methods*. Coeliac disease patients were evaluated in two different groups based on serum endomysial antibody (EmA) titers (EmA (+) and EmA (−)), and the data obtained by conventional and TDE studies were compared between the patient groups and healthy controls. *Results*. There was no significant difference between EmA (+) and EmA (−) groups in terms of the conventional TTE parameters, including ejection fraction (EF), fractional shortening (FS), and left ventricle end diastolic diameter (LVEDD), that show the left ventricular systolic function (*P* = 0.727, *P* = 0.317, *P* = 0.118). TDE showed a significant difference in left ventricle (LV) isovolumic relaxation time (LV IVRT) and LV myocardial performance index (LV MPI) parameters between EmA (+) and EmA (−) patient groups (*P* < 0.0001). *Conclusion*. The measurement of LV MPI and LV IVRT parameters by TDE would be beneficial in early determination of the cardiac involvement and establishing appropriate treatment and followup of patients with coeliac disease as well as in making distinction between EmA (+) and EmA (−) patients.

## 1. Introduction

Coeliac disease (CD) is childhood disorder characterized by malabsorption and steatorrhoea but can also affect adults of any age [1, 2]. Studies have shown that Coeliac disease affects about 1% of European and American children and adults [3, 4]. This disease may present in various forms depending on the age at onset and disease duration and may be silent or remain asymptomatic [5, 6]. Autoimmune myocarditis and idiopathic dilated cardiomyopathy are a well-known cause of significant morbidity and mortality among comorbidities of Coeliac disease [7]. In Coeliac disease, many theories have been proposed to explain the development of cardiomyopathy [8, 9]. One theory suggests that intestinal malabsorption leads to nutritional deficiency, and another theory suggests that abnormalities of intestinal absorption leads to increased intestinal absorption of antigens and infectious agents and thus to activation of immune mechanisms, which eventuates in myocardial damage. Finally, the direct immune response may cause damage to small intestine and myocardium [10]. In one study, gluten-free diet was found to be protective in the development of autoimmune diseases [11]. However, it is controversial whether gluten-free diet prevents the progression once the Coeliac disease has been diagnosed [12]. Tissue Doppler echocardiography (TDE) is widely accepted to be beneficial in determining subclinical ventricular functions in regurgitant valvular diseases, anthracycline cardiotoxicity, the early stage of cardiomyopathies, and cardiac transplant rejection [13–16]. A small number of studies have identified the role of TDE in Coeliac disease [17]. In the literature, there are limited data about TDE studies investigating the effects of the treatment with gluten-free diet on subclinical cardiac dysfunction accompanying to Coeliac disease in children. We hypothesized that identification and treatment of patients with Coeliac disease before dilated cardiomyopathy and heart failure symptoms occur would play an important role in reducing cardiac morbidity and mortality of these patients.

For this purpose, endomysial antibody (EmA) positive and EmA negative patients underwent conventional and tissue Doppler echocardiography studies and measurements compared with the healthy control group subjects. 

## 2. Methods

Seventy-five children (25 male (34%) and 50 female (66%); mean age 9.3 ± 4.6 years; range 5 months–19 years), who were previously diagnosed as having CD were enrolled in this study at Diskapi Children's Research Hospital between May 2009 and June 2010. Mean follow-up time was 34 ± 15.1 months. In the study group, patients were divided into two groups based on serum EmA titer on echocardiographic examination. Control group consisted of 30 age-matched healthy children. In the serum IgA antiendomysial antibody assay, EmA values were determined with a commercially available ELISA indirect immunofluorescence method (Euroimmun, Germany), as previously described [18]. All sera manifesting fluorescence titer ≥1 : 20 were considered to be positive. In the gastrointestinal endoscopy studies, patients underwent upper gastrointestinal endoscopy with at least three biopsy in the descending duodenum by EG 450PE5 (Fujinon, Japan). Histological findings were described using the modified Marsh classification [19]. Histopathologic examination of duodenal biopsies of all patients revealed shortening of the villi, crypt hyperplasia, and an increased number of intraepithelial lymphocytes (type 3) according to modified Marsh classification. In the Echocardiographic Examination, all patients were examined in a semisupine left-lateral position by one observer. Echocardiographic imaging was performed using a Vivid-7 machine (GE; Vingmed, Hovik, Norway), equipped with 3 and 7 MHz transducers and all patients had continuous electrocardiographic monitoring. Routine echocardiographic examinations were performed before conventional and tissue Doppler echocardiography to eliminate any congenital or acquired heart disease. Conventional echocardiographic measurements were performed in accordance with the American Society of Echocardiography's Guidelines [20]. Images were obtained in the parasternal long axis and apical four-chamber views. Left ventricle ejection fraction was estimated from M-mode tracings, using the Teichholz formula [21]. The tricuspid and mitral valve Doppler signals were recorded in the apical four-chamber view, with the Doppler sample volume placed at the tip of the valves. Peak early filling velocity (E wave), peak atrial systolic velocity (A wave), early-to-late diastolic flow ratio (E/A), deceleration time (DT), isovolumetric relaxation time (IVRT), and isovolumic contraction time (IVCT) were measured for tricuspid valve (TV) and mitral valve (MV). Tissue Doppler echocardiography (TDE) was performed from the apical four-chamber view. Myocardial velocity profiles of the lateral tricuspid annulus and lateral mitral annulus were obtained by placing the sample volume at the junction of the tricuspid annulus and the right ventricle (RV) free wall and at the junction of the mitral annulus and LV posterior wall, respectively. With this modality, the values recorded were the early (E′) and late (A′) diastolic mitral and tricuspid annular velocities, and the ratio of E′/A′. Right ventricle and left ventricle myocardial performance index (MPI) was obtained by dividing the sum of isovolumic relaxation time (IVRT) and isovolumetric contraction time (IVCT) by the ejection time (ET) (MPI = (ICT + IVRT)/ET). All patients underwent an echocardiographic examination that was performed by the same observer blind to the retrospective review of patient records of all children. The protocol was approved by the Local Research Ethics Committee, and all subjects gave informed consent.

### 2.1. Statistical Analysis

The normality of each variable was tested with the Shapiro-Wilk test of Normality. Since the distributions in groups were significantly different from normality, nonparametric hypothesis tests were applied throughout the whole analysis. Mann-Whitney *U* test was used to investigate the differences between two study groups: patients versus Controls. The patients were then evaluated for their serum EmA titers and divided into two subgroups: EmA (+) and EmA (−). In this case, Kruskal-Wallis analysis of variance was applied to evaluate the differences between three groups. In case of statistical significance, Mann-Whitney *U* tests were used to further assess pairwise differences. Bonferroni correction was applied, as well. Pearson chi-square test was used for testing categorical variables. Alpha value of 0.05 was accepted as the level of significance. All statistical analyzes were carried out with SPSS (version 18.0) statistical software. 

## 3. Results

Seventy-five children (25 male (34%) and 50 female (66%)) were diagnosed at a mean age of 9.3 ± 4.6 years; range 5 months to 19 years. Mean follow-up time was 34 ± 15.1 months. Demographic and laboratory findings of groups are given in Table 1. While there was no difference between EmA (+) and EmA (−) groups in terms of age, gender, weight, height, and age at diagnosis, a significant difference was found in mean follow-up time (34.2 ± 28.1 months in EMA (+) group and 20.6 ± 18.8 months in EMA (−) group). Conventional echocardiography indices of patients and control group were shown in Table 2. All parameters were statistically significant between patient and control groups. Interventricular septal systolic dimension (IVSD) and left ventricle end diastolic diameter (LVEDD) parameters showed significant difference between EmA (+) and EMA (−) groups and the control group (*P* < 0.0001). Ejection fraction (EF) and fractional shortening (FS) parameters, which are commonly used in assessing left ventricle (LV) systolic function in clinical practice, showed no significant difference between groups. There was no significant difference in pulsed wave (PW) Doppler echocardiography parameters including MV E, MV A, MV E/A, and TV E between EmA (+) and EmA (−) groups, while these parameters showed significant difference between the control group and the other groups (*P* < 0.0001). A significant difference was found between EmA (+) group and the control group in terms of MV IVRT and TV IVCT parameters (*P* = 0.001, *P* = 0.019). Left ventricular myocardial performance index showed a significant difference between EmA (+) and EmA (−) groups and the control group (*P* < 0.0001), but not between EmA (+) and EmA (−) groups. The tissue Doppler echocardiographic parameters are given in Table 3. MV E′, MV A′, TV E′, TV MPI parameters showed no significant difference between EmA (+) and EmA (−) groups, whereas a significant difference between patient groups and the healthy control group (*P* < 0.0001). There were significant differences in MV S′, MV IVCT, TV A′, and TV IVRT parameters between EmA (+) and EmA (−) patients groups and the healthy control group (*P* = 0.033, *P* = 0.034, *P* = 0.020, *P* = 0.001, resp.). Only MV IVRT and MV MPI parameters showed significant difference between EmA (+) and EmA (−) patient groups (*P* < 0.0001).

## 4. Discussion

The clinical spectrum of coeliac disease (CD) continues to evolve. It is a rare disorder effecting young children with a range of symptoms ranging from asymptomatic to severely affected. The classic presentation is failure to thrive, malnutrition, diarrhea, abdominal pain, and distension, but in recent years, because of atypical presentations, the term referred to as the “coeliac disease iceberg” has entered to the literature [17, 22]. An increased incidence of dilated cardiomyopathy has been reported in CD patients [23–25]. Lodha et al. [26] have reported a case of dilated cardiomyopathy with symptomatic CD. A study of 52 patients reported an incidence of 5.8% for coeliac disease in patients with dilated cardiomyopathy [27]. In our study, none of the patients had any degree of dilated cardiomyopathy diagnosed with echocardiography. Our results suggest that there was no significant difference in age, heart rate, and systemic blood pressure parameters between the patient groups and the healthy control group. Gender distribution showed no significant difference between EmA (+) and EmA (−) groups. Weight and height differences did not reach the statistical significance level between the patient groups, and these parameters were found to be significantly lower in the patient groups than healthy controls. Mean followup time was 20.6 ± 18.8 and 34.2 ± 28.1 months in EmA (+) and EmA (−) groups, respectively, and this difference reached statistical significance level (*P* < 0.05). 

Makhdoom and Randall [28] established a relationship between gluten-free diet and negative serum EmA test and the cardiac functions. In our study, the correlation between the length of followup and negative serum EmA test is consistent with the literature. Among laboratory parameters, significantly lower mean corpuscular volume (MCV) and higher red blood cell distribution width (RDW) values were found in EmA (+) patients than EmA (−) patients, which were interpreted as the expected findings in Coeliac disease. However, although conventional and tissue Doppler echocardiography parameters of the patients included in the present study were within normal limits, there was statistically difference between the patient groups and the control group, which suggested that subclinical cardiac dysfunction has developed. Prati et al. [25] have confirmed that patients with end-stage heart failure are at increased risk for coeliac disease as compared to the general population. De Bem et al. [29] showed a prevalence of 2.7% for coeliac disease in South Brazilian precardiac transplant patients with advanced cardiomyopathy. Frustaci et al. [30] showed a higher prevalence of CD among patients with idiopathic congestive heart failure. Thirteen of the 187 patients with biopsy-proven myocarditis had CD-related autoantibodies, nine (4.4%) of whom also had positive serum EmA titers. In our study, EF and FS parameters, which are commonly used in the assessment of LV systolic function by conventional evaluation of transthoracic echocardiography (TTE), showed no significant difference between groups (Table 2). IVSD and LVEDD parameters showed significant difference between patients and controls. Although MV E, MV A, MV E/A, TV E, and TV E/A parameters, which are used in the evaluation of diastolic functions by conventional TTE, showed a significant difference between patients and control group, no evidence of diastolic dysfunction was found in all groups. There was a significant difference in MV IVRT and TV IVCT parameters between EmA (+) patients and the control group. Therefore, because of giving early information about cardiac functions, TDE may be useful to obtain evidence of subclinical impairment of ventricular function. There are few published studies in the pediatric age group with Coeliac disease [31, 32]. Although our results showed a significant difference between the patient and control groups in terms of MV E′ and MV A′ among tissue Doppler echocardiographic parameters of left ventricle, no evidence of diastolic dysfunction was observed. However, there was a significant difference between EmA (+) and EmA (−) patient groups in terms of MV MPI as a marker of left ventricular dysfunction, with a higher MV MPI value in EmA (+) patients. There was significant difference between patients and controls in terms of TV E′ and TV MPI among right ventricular tissue Doppler echocardiographic parameters, whereas there is no significant difference between EmA (+) and EmA (−) patient groups. In Polat et al.'s [31] study, a significant difference was found in MV S′ parameters between EmA (−) patients with Coeliac disease and the healthy control group. Our findings are compatible with the literature and showed significant difference for MV S′ between EmA (+) and EmA (−) patients and the control group (*P* = 0.033). According to our study, both with conventional echocardiography and with TDE indices, we observed significant differences between CD patients and control group. However, MV IVRT and MV MPI parameters were approved to have the ability to differentiate between EmA (+) and EmA (−) patients. In particular, the striking point is that there was no statistically significant difference between CD patients and the control group in terms of ejection fraction and fractional shortening parameters that are the indicators of the LV systolic function. But prolonged IVRT and MV MPI measured with TDE may be a marker of early cardiac dysfunction. Considering all these data, to diagnose and treat childhood coeliac disease is important for the future of the patients.

*Study Limitations.* Lack of data concerning long-term followup and progression of the patient group is the most important limitation of our study. Another limitation is that a multicenter study involving more patients is needed to establish the relationship between the response of these patients to treatment and signs of dilated cardiomyopathy and heart failure. Followup of all patients will continue to determine the results of long-term followup of the patients and whether the symptoms of manifest dilated cardiomyopathy will develop.

## 5. Conclusion

The present study suggests that TDE is better to identify subclinical early stage of cardiac changes in patients with CD. We recommend to use MV MPI and IVRT parameters that are measured by tissue Doppler echocardiography in differentiating between EmA (+) and EmA (−) patients and therefore monitoring the effectiveness of treatment during the followup of patients with Coeliac disease.

## Figures and Tables

**Table 1:** Demographic data of the groups.

	Group 1 EmA (+) (*n*: 26)	Group 2 EmA (−) (*n*: 49)	Control (*n*: 30)
Age (months)	109.6 ± 56.6^d^	111.9 ± 55.2^d^	116.53 ± 48.58^d^
Sex (male/female)	18/26^d^	7/49^d^	17/30^b,c^
Weight (kg)	30.7 ± 13.2^d,e^	34.4 ± 14.2^d,f^	50.93 ± 13.18^e,f^
Height (cm)	122.8 ± 28.3^d,e^	126.0 ± 27.5^d,f^	158.03 ± 12.75^e,f^
Heart rate (beat/minute)	91.0 ± 8.1^d^	91.1 ± 15.8^d^	91.50 ± 16.11^d^
Arterial tension systolic (mmHg)	96.6 ± 7.1^d^	94.1 ± 7.4^d^	95.33 ± 8.19^d^
Arterial tension diastolic (mmHg)	52.1 ± 7.6^d^	50.1 ± 6.5^d^	52.00 ± 5.19^d^
Age at diagnosis (months)	88.6 ± 49.6^d^	79.9 ± 49.8^d^	—
Mean follow up period (months)	20.6 ± 18.8^a^	34.2 ± 28.1^a^	—
Hb (g/dl)	12.2 ± 1.8	12.7 ± 1.3	—
MCV (fl)	76.4 ± 7.6^a^	80.2 ± 5.9^a^	—
MCH	26.0 ± 3.3	27.1 ± 2.4	—
MCHC	33.2 ± 1.7	33.7 ± 1.5	—
RDW	14.6 ± 2.2^a^	13.4 ± 2.1^a^	—
Ferritin (ng/mL)	17.3 ± 15.4	20.1 ± 16.1	—

^a^Group 1 and group 2 statistically significant (*P* < 0.05).

^b^Group 1 and control group statistically significant (*P* < 0.05).

^c^Group 2 and control group statistically significant (*P* < 0.05).

^d^Group 1 and group 2 statistically not significant (*P* > 0.05).

^e^Group 1 and control group statistically significant (*P* < 0.001).

^f^Group 2 and control group statistically significant (*P* < 0.001).

Hb: hemoglobin, Htc: hematocrit, MCV: mean corpuscular volume, MCH: mean corpuscular hemoglobin, MCHC: mean corpuscular hemoglobin concentration, RDW: red blood cell distribution.

**Table 2:** Conventional echocardiography indices of groups.

	Group 1 EmA (+) (*n*: 26)	Group 2 EmA (−) (*n*: 49)	Control *n* = 30	*P*
IVSS	10.0 ± 2.60^b^	9.5 ± 2.14^d^	6.8 ± 0.35^b,d^	<0.0001
IVSD	7.1 ± 1.19	7.9 ± 1.37	7.2 ± 0.23	0.402
LVEDD	33.4 ± 7.93^b^	33.7 ± 8.98^d^	28.9 ± 0.66^b,d^	<0.0001
LVEDS	21.6 ± 3.83	22.4 ± 3.84	20.1 ± 0.88	0.118
FS	31.5 ± 5.29	33.2 ± 7.39	31.7 ± 0.87	0.317
EF	63.4 ± 8.08	65.0 ± 7.92	64.3 ± 1.34	0.727
MV E	1.0 ± 0.14^b^	1.0 ± 0.16^d^	1.7 ± 0.05^b,d^	<0.0001
MV A	0.6 ± 0.16^b^	0.6 ± 0.17^d^	0.4 ± 0.04^b,d^	<0.0001
MV E/A	1.8 ± 0.38^b^	1.7 ± 0.43^d^	1.0 ± 0.07^b,d^	<0.0001
LV IVRT	108.9 ± 35.46^a^	98.6 ± 29.65	85.8 ± 8.31^a^	0.001
LV IVCT	86.0 ± 22.98	76.2 ± 21.45	78.8 ± 4.57	0.464
TV E	0.7 ± 0.12^b^	0.7 ± 0.17^d^	0.5 ± 0.04^b,d^	<0.0001
TV A	0.6 ± 0.15	0.5 ± 0.16	0.5 ± 0.05	0.716
TV E/A	1.2 ± 0.32^b^	1.4 ± 0.42^d^	0.8 ± 0.15^b,d^	<0.0001
RV IVRT	92.1 ± 29.21^b^	86.9 ± 31.79^d^	59.0 ± 6.57^b,d^	<0.0001
RV IVCT	78.5 ± 28.13^a^	74.5 ± 25.41^c^	65.9 ± 1.39^a,c^	0.019

^a^Group 1 and control group statistically significant (*P* < 0.05).

^b^Group 1 and control group statistically significant (*P* < 0.001).

^c^Group 2 and control group statistically significant (*P* < 0.05).

^d^Group 2 and control group statistically significant (*P* < 0.001).

EF: ejection fraction, FS: fractional shortening, IVSD: interventricular septal diastolic dimension, IVSS: interventricular septal systolic dimension, IVRT: isovolumic relaxation time, IVCT: isovolumic contraction time, LV: left ventricle, LVEDD: left ventricle end diastolic diameter, LVEDS: left ventricle end systolic diameter, MV E: mitral valve early diastolic velocity, MV A: mitral valve late diastolic velocity, RV: right ventricle, TV E: tricuspid valve early diastolic velocity, TV A: tricuspid valve late diastolic velocity.

**Table 3:** Tissue Doppler echocardiography indices of groups.

	Group 1 EmA (+) (*n*: 26)	Group 2 EmA (−) (*n*: 49)	Control *n* = 30	*P*
MV E′	0.16 ± 0.03^b^	0.16 ± 0.04^d^	0.11 ± 0.01^b,d^	<0.0001
MV A′	0.10 ± 0.12^b^	0.08 ± 0.07^d^	0.04 ± 0.02^b,d^	<0.0001
MV E′/A′	2.37 ± 0.71	3.02 ± 4.63	3.31 ± 5.42	0.616
MV S′	6.85 ± 1.32^a^	6.65 ± 1.32^c^	7.53 ± 0.49^a,c^	0.033
MV IVRT	70.46 ± 24.35^e^	58.96 ± 8.82^e^	57.13 ± 2.89	<0.0001
MV IVCT	66.96 ± 26.99^a^	63.55 ± 21.11^c^	52.13 ± 3.66^a,c^	0.034
LV MPI	1.02 ± 0.44^b,e^	0.68 ± 0.25^d,e^	0.56 ± 0.07^b,d^	<0.0001
TV E′	0.39 ± 0.95^b^	0.19 ± 0.12^d^	0.41 ± 0.02^b,d^	<0.0001
TV A′	0.14 ± 0.12^a^	0.14 ± 0.11	0.11 ± 0.02^a^	0.020
TV E′/A′	1.30 ± 0.43	1.71 ± 0.92	1.34 ± 0.17	0.901
TV S′	6.50 ± 1.56	7.12 ± 3.95	7.06 ± 0.59	0.115
TV IVRT	72.35 ± 26.01^a^	75.24 ± 26.80^c^	47.13 ± 5.85^a,c^	0.001
TV IVCT	55.88 ± 15.39	66.47 ± 18.82	54.30 ± 1.66	0.715
RV MPI	0.86 ± 0.27^b^	0.77 ± 0.22^d^	0.54 ± 0.02^b,d^	<0.0001

^a^Group 1 and control group statistically significant (*P* < 0.05).

^b^Group 1 and control group statistically significant (*P* < 0.001).

^c^Group 2 and control group statistically significant (*P* < 0.05),

^d^Group 2 and control group statistically significant (*P* < 0.001).

^e^Group 1 and group 2 statistically significant (*P* < 0.001).

IVRT: isovolumic relaxation time, IVCT: isovolumic contraction time, LV: left ventricle, MPI: myocardial performance index, MV E′: mitral valve early diastolic velocity, MV A′: mitral valve late diastolic velocity, MV S′: mitral valve systolic velocity, RV: Right ventricle, TV E′: tricuspid valve early diastolic velocity, TV A′: tricusp valve late diastolic velocity, TV S′: tricuspid valve systolic velocity.
